# Intermolecular Interaction in Methylene Halide (*CH*_2_*F*_2_, *CH*_2_*Cl*_2_, *CH*_2_*Br*_2_ and *CH*_2_*I*_2_) Dimers

**DOI:** 10.3390/molecules24091810

**Published:** 2019-05-10

**Authors:** László Almásy, Attila Bende

**Affiliations:** 1 State Key Laboratory of Environment-friendly Energy Materials, Southwest University of Science and Technology, Mianyang 621010, China; 2 Institute for Solid State Physics and Optics, Wigner Research Centre for Physics, Konkoly Thege út 29-33, 1121 Budapest, Hungary; 3 Molecular and Biomolecular Physics Department, National Institute for Research and Development of Isotopic and Molecular Technologies, Donat Street, No. 67-103, Ro-400293 Cluj-Napoca, Romania

**Keywords:** methylene halide, intermolecular interaction, halogen bonding, SAPT, molecular liquid

## Abstract

The intermolecular interaction in difluoromethane, dichloromethane, dibromomethane, and diiodomethane dimers has been investigated using high level quantum chemical methods. The potential energy curve of intermolecular interaction along the C⋯C bond distance obtained using the coupled-cluster theory with singles, doubles, and perturbative triples excitations CCSD(T) were compared with values given by the same method, but applying the local (LCCSD(T)) and the explicitly correlated (CCSD(T)-F12) approximations. The accuracy of other theoretical methods—Hartree–Fock (HF), second order Møller–Plesset perturbation (MP2), and dispersion corrected DFT theory—were also presented. In the case of MP2 level, the canonical and the local-correlation cases combined with the density-fitting technique (DF-LMP2)theories were considered, while for the dispersion-corrected DFT, the empirically-corrected BLYP-D and the M06-2Xexchange-correlation functionals were applied. In all cases, the aug-cc-pVTZ basis set was used, and the results were corrected for the basis set superposition error (BSSE) using the counterpoise method. For each molecular system, several dimer geometries were found, and their mutual orientations were compared with the nearest neighbor orientations obtained in recent neutron scattering studies. The nature of the intermolecular interaction energy was discussed.

## 1. Introduction

Weak intermolecular interactions play important roles in a wide range of chemical and biological processes at the supramolecular level. These supramolecular systems are generally governed by different types of intermolecular interactions, like hydrogen bonds (H-bonds) [1], weak van der Waals (vdW) forces [2], or charge-transfer complexes [3]. Attractive interactions between aromatic π systems are one of the most studied noncovalent vdW forces responsible for many supramolecular organization and recognition processes. They are one of the most important interactions in the vertical base stacking of DNA [4,5] and also could influence the tertiary structure of proteins [6]. However, less is said about other weak vdW forces that have almost the same nature of attractive interaction, but they are formed between saturated hydrocarbons [7,8] or molecular systems that contain halogen atoms [9,10,11,12]. Studies of these interactions have become increasingly important, and many authors point out their special role in mediation of protein–protein, protein –nucleic acid, and receptor–ligand recognition and binding of molecular systems containing halogen atoms (for examples, see [13,14] and the references therein).

Interactions between molecules containing halogen atoms are generally called *halogen bonds*, and they were the subject of many theoretical [15,16,17,18,19] and experimental [20,21,22,23,24] studies and are discussed in many review articles [25,26,27,28]. Initially, the *halogen bonding* [29,30] was considered as a charge transfer effect from an electron-donating negatively-charged atom to the neighboring molecule to the $\sigma^{*}$ orbital of the covalent bond (X–Y) defined by a halogen atom (X) and the “holder” atom (Y, e.g., a carbon atom) [31,32]. However, an increasing number of theoretical investigations have highlighted the electrostatic character of these *halogen bonds* [18,33,34], particularly the role of the atomic quadrupolar effect [35] in the intermolecular electrostatic interaction. On the other hand, the behavior of the various halogen atoms (F, Cl, Br, or I) in different electron donor-acceptor or charge-transfer supramolecular complexes is still under debate [36,37], as well as the physical origin of these molecular interactions. For example, Osuna et al. [38] found that the relative orientation of the C–F⋯F bond affects the bond strength, while Price et al. [39] proposed an anisotropic model built by a combination of the repulsion, dispersion, and electrostatic forces in the case of –Cl⋯Cl– interaction. A comparative study on the nature and strength of weak hydrogen bonding between the C(sp${}^{3}$)–H, C(sp${}^{2}$)–H, and C(sp)–H donor bonds and F–C(sp${}^{3}$) acceptors was presented by Grimme’s group [40]. One of their relevant conclusions was that double-zeta quality was not appropriate for the investigation of these weakly-bonded systems, but well-balanced basis sets of at least TZVPP quality are needed. Another important observation by them was that in most of the studied molecular structures, the dispersion interaction term dominated the entire attraction. Joining together all these findings about halogen–halogen and halogen–hydrogen intermolecular interactions, one can conclude that the nature of these interaction shows a complicated picture where many different physical effects, like electrostatic, exchange repulsion, permanent dipole-dipole, or induced dipole-dipole contribute to the final magnitude of the intermolecular interaction energy (IIE) [41,42,43,44].

With two hydrogen and two halogen atoms attached to the same carbon atom, methylene halides ($CH_{2}X_{2}$, X=F,Cl,Br, or *I*) can act either as proton donors or proton acceptors, and thus, they can easily form hydrogen-bonded dimers. In the classical H-bond system (X–H⋯Y), the X–H (proton donor) bond length increases, and the $\nu_{X–H}$ stretching band undergoes a red shift upon formation of a hydrogen bond. However, studies of Hobza et al. revealed an interesting new class of H-bonds when a halogen atom is present in the molecule. This is called an “improper H-bond”, and one of the main characteristic of them is the blue shift of the $\nu_{X–H}$ stretching frequencies [45,46,47,48]. The nature of the C–H⋯F–C bond in different fluoromethanes was studied by Kryachko et al. [49] and Ebrahimi et al. [50]; these studies demonstrated the improper H-bond character of the C–H proton donor induced by the presence of the fluorine atom. A systematic analysis of these “improper H-bonds”, for the molecular class of $CH_{2}X_{2}$, which would include also chlorine, bromine, and iodine atoms, could not be found in the literature. However, such analysis was performed by Zierkiewicz et al. for molecular families of $CX_{3}H$ and XH, (X=F, Cl, Br, or I) in interaction with water [51]. They found that the characteristics of bonding in the hydrogen halide complexes correspond to the standard H-bonding (an elongation of the XH bond and red shift of its stretch frequency), whereas those in the $CX_{3}H\cdots OH_{2}$ complexes (X = F, Cl) are typical of improper blue-shifting H-bonding (a contraction of the CH bond and blue shift of the respective stretching frequency). This finding suggests to us that the nature of the *halogen bond* depends basically on the particularity of the donor and acceptor molecules.

In this paper, we study the series of methylene halide dimers with various theoretical methods and also relate their interactions to the closest neighbor structures in the liquid phase, as obtained from previous diffraction experiments. After a general description of the used theoretical methods, we present the possible dimer configurations for each methylene halide. This is followed by the comparison of the potential energy curves considering different high-level electron correlation methods for the strongest dimer configuration of each methylene halide with the $C_{s}$ common symmetry group. We discuss the nature of the intermolecular interaction in methylene halides taking into account the H⋯X, H⋯H, and X⋯X pair interactions. In the final section, we compare the computed dimer structures with the most frequent closest neighbor configurations observed in the liquid phase.

## 2. Computational Methods

For the computation of intermolecular interactions, local (L) electron correlation methods [52,53,54] at the second order perturbation theory level have been proven to give values that are very close to the standard Møller–Plesset perturbation theory (MP2) results, and by construction, they are virtually free of the basis set superposition error (BSSE) [53,54]. Linear scaling of the computational cost as a function of the system size [55] makes it possible to treat larger systems or to use larger basis sets. Using the density fitting (DF) approximation of the electron repulsion integrals [56,57,58], one can reduce again the computation time by about one order of magnitude, applying it both in the Hartree–Fock (HF) and LMP2 cases (DF-HF and DF-LMP2). In particular, the efficiency of DF-LMP2 method was clearly demonstrated by Hill et al. [59] in describing the π–stacked intermolecular interaction dominated by the dispersion forces in the case of the stacking benzene dimer structure. In this way, the computational cost was reduced to O(N) – $O\left(N^{2}\right)$ without losing much in accuracy compared with the case of the conventional second-order Møller–Plesset perturbation theory (MP2), which scales formally with the order of $O\left(N^{5}\right)$. In spite of the fact that the local correlation treatment combined with the density-fitting technique can, in general, provide calculations with much lower computational costs, the deficiency of the standard MP2 theory (for example, in π–stacked systems, the LMP2 method overestimates the dispersion forces) remains also an attendant of the LMP2 method. Hill et al. [59] performed a detailed theoretical investigation for different dimer configurations of benzene, and they found that the LMP2 results are quite far from the counterpoise corrected CCSD(T)values. At the same time, when the so-called spin-component scaled (SCS) MP2 theory [60] was applied (both for canonical and localized orbitals), the discrepancy was small compared with the CCSD(T) results. In a later work [61], they have shown that in case of stacked nucleic acid systems, the SCS-LMP2 method fails to describe the IIE correctly, where its mean deviation from the best estimated values of the S22set [62] is −1.62 kcal/mol (usually, it overestimates the interaction energy). Accordingly, instead of the default scaling factor of 6/5 for antiparallel spins and 1/3 for parallel spins, they completely neglected the contribution from antiparallel-spin electron pairs to the MP2 energy and scaled the parallel contribution by 1.76. In this way, they obtained a mean deviation of −0.04 kcal/mol. The method is called spin-component scaled LMP2 for nucleobases (SCSN-LMP2). Choosing the spin-component scaling factors empirically, Hill et al. [59] emphasized that this methodology can no longer be considered as a truly ab initio method, since it provides only a substantial correction to the MP2 overestimation of the dispersion energy at no extra cost. To exclude the BSSE effects in the case of the conventional MP2 method, the Boys–Bernardi counterpoise correction scheme was used [63].

In the past few years, important efforts have been made to develop new, efficient approximation techniques in order to reduce the computational cost of the high electron correlation methods. Good results were obtained by introducing the explicitly-correlated $R_{12}$ theories in the electron correlation [64,65,66]. These theories bypass the slow convergence of conventional methods, by augmenting the traditional orbital expansions with a small number of terms that depend explicitly on the interelectronic distance $r_{12}$. Modern $R_{12}$ (or $F_{12}$) methods [67] can deliver MP2 energies that are converged to chemical accuracy (1 kcal/mol) in triple- or even double-zeta basis sets and can also provide high accuracy much faster than the conventional methods. We used the F12bcomputing scheme [67] for the CCSD(T)-F12 method, considering the fixed amplitude ansatz ($T_{ij}^{ij,1}$ = 1/2 and $T_{ij}^{ij,-1}$ = 1/4) and Slater-type frozen geminals [68]. The aug-cc-pVTZ was considered as the auxiliary basis set for the density-fitting and RI expansion.

The supramolecular ab initio calculations have become very popular tools for the investigation of intermolecular interactions, especially when using the above-presented new approximation techniques. However, these methods do not provide information on the character of these interactions. To get more insight into the physical nature of the intermolecular interaction, we have used the interaction energy decomposition scheme as the symmetry-adapted perturbation-theory (SAPT) method [69] with energy components up to the second order in *V*. A detailed presentation of the SAPT theory is described in [69], and a brief description of the SAPT theory was also given in our previous work [70].

Using the DF-LMP2 method implemented in the Molpro program package suite [71], we have performed geometry optimization for different dimer conformations of methylene halide ($CH_{2}F_{2}$, $CH_{2}Cl_{2}$, $CH_{2}Br_{2}$, and $CH_{2}I_{2}$) dimers considering the aug-cc-pVTZ basis set [72,73,74,75,76]. Taking the program parameter descriptions as presented in [59], we used the following input settings: (i) we considered the Pipek–Mezey (PM) localization procedure [77]; (ii) in order to solve an occurrent poor orbital localization in the PM technique, when the larger diffuse basis set were used, we eliminated the contribution of the diffuse basis functions to the localization criteria by setting the corresponding rows and columns of the overlap matrix used in the PM localization to zero. Considering the local character of the occupied and virtual orbitals in the local correlation treatment, one can easily obtain also the dispersion part (an intermolecular effect) of the correlation contribution [78].

The intermolecular potential energy curves for methylene halide dimers for several C⋯C bond distances were computed using different theoretical models. Accordingly, the Truhlar’s M06-2x and the dispersion-corrected BLYP-D results were compared with the density-fitting HF, with the density-fitting local correlation (DF-LMP2 and DF-LCCSD(T)), as well as with the explicitly-correlation (CCSD(T)-F12) methods, where the M06, M06-2X [79], and DFT-D(considering the BLYP-D XCfunctional with empirical dispersion corrections [80,81]) energy values were obtained using the NWChem program package [82]. It was shown by Bauzá et al. [83] that most of the hybrid and pure DFT functionals largely overestimate the interaction energies in halogen bonding complexes, and only the M06-2X DFT functional gives reasonably good results compared with the CCSD(T)/aug-cc-pVTZ reference method. The potential energy curves are compared also with two reference methods considering the canonical MP2 and the coupled-cluster with singles, doubles, and perturbative triples excitation CCSD(T) theories, as well as with SCSN-MP2 method, all of them implemented in the same Molpro program package suite [71]. The intermolecular energy decomposition was performed using the SAPT theory implemented in the Molpro package [84]. Molecular structures were visualized and analyzed using the open source Gabedit molecular graphics program [85].

## 3. Intermolecular Interactions and Dimer Structures

**Figure 1:**
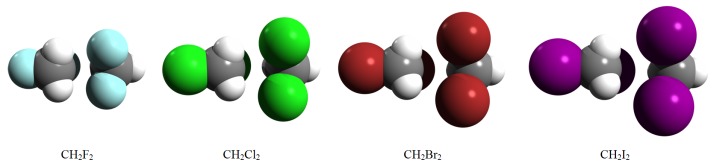
Selected configurations for methylene halide dimers considering their atomic volumes.

Throughout the following section, we present a detailed theoretical analysis for methylene halide ($CH_{2}F_{2}$, $CH_{2}Cl_{2}$, $CH_{2}Br_{2}$, and $CH_{2}I_{2}$) dimers describing their intermolecular interactions and dimer configurations. A suite of a selected common configuration for methylene halide dimers considering their atomic volumes is presented in Figure 1.

**Figure 2:**
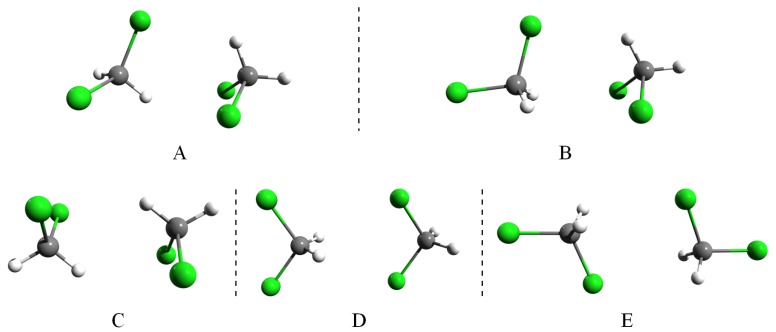
Relative orientation of the methylene halide monomers in the five stable dimer configurations (**A**—asymmetric; **B**—plane-symmetric; **C** and **D**—with C${}_{2h}$ point group symmetry and **E**—with C${}_{2v}$ point group symmetry).

The geometry optimizations were done using the DF-LMP2 level of theory considering the aug-cc-pVTZ basis set. We have found several geometry configurations for each methylene halide dimer: three bounded dimers for $CH_{2}F_{2}$ and five bounded structures for $CH_{2}Cl_{2}$, $CH_{2}Br_{2}$, and $CH_{2}I_{2}$. Since these dimer structures are very similar for different methylene halides, we denote them with *A*, *B*, *C*, *D*, and *E*. Configuration *A* is totally asymmetric ($C_{1}$); *B* belongs to the $C_{s}$ point symmetry group with a single symmetry plane; *C* and *D* belong to the $C_{2h}$ with a $C_{2}$ rotation axis and a symmetry plane with inversion center; while *E* belongs to $C_{2v}$ symmetry group having a simple $C_{2}$ rotation axis with two symmetry planes. The graphical view of the five conformations is shown in Figure 2. Since, for the $CH_{2}F_{2}$ case, the *A* and *C* dimer configurations are missing (they converged to *B*), we computed and compared the potential energy curves for the *B*-type dimer configuration for all methylene halide systems.

### 3.1. Methylene Chloride ($CH_{2}Cl_{2}$)

In the case of methylene chloride (or dichloromethane), all five dimer configurations were found. The strongest bounded dimer was *A*, while the weakest one was the *E* configuration. For the five methylene chloride dimers, the interaction energy diminishes in alphabetic order. The interaction energies at the HF, LMP2, and LCCSD(T) levels of theory, as well as the C⋯C bond distances and dipole moments were presented in our previous work [86]. Here, we continue to characterize the intermolecular interaction of the *B* dimer considering further theoretical methods such as the coupled-cluster theory with singles, doubles, and perturbative triples excitation CCSD(T) and its explicitly-correlated approximations (CCSD(T)-F12), the canonical MP2 with BSSE correction, the spin-component scaled MP2 for nucleobases, and the dispersion corrected DFT (empirically-corrected BLYP-D and M06-2X exchange-correlation functionals) methods and compare the results obtained by them. The CCSD(T) theory was taken as a reference for comparison with the other theoretical methods.

**Figure 3:**
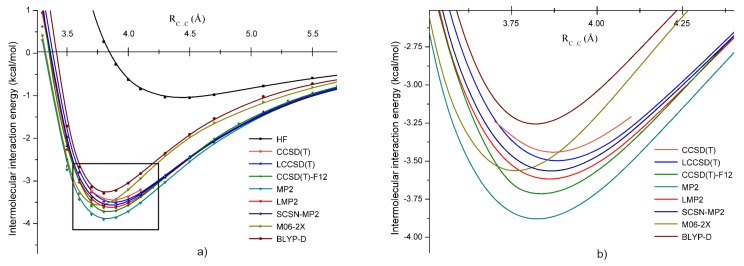
Potential energy curves for the *B* form of the dichloromethane dimer obtained at the HF, CCSD(T), LCCSD(T), CCSD(T)-F12,Møller–Plesset perturbation (MP2), LMP2, SCSN-MP2, M06-2X, and BLYP-D levels of theory using the aug-cc-pVTZ basis set; (**a**) for larger intermolcular C⋯C bond separation (3–6 Å) and (**b**) around the equilibrium C⋯C distance (3.5–4.5 Å).

The potential energy curves obtained with these methods are shown in Figure 3. Generally, one can conclude that all applied methods gave bounded structures for the *B* dimer geometry of $CH_{2}Cl_{2}$. In the first step, the coupled-cluster methods were compared, where, in addition, two different approximations of explicitly- (F12) and local-correlation (L) were also considered. Since the canonical CCSD(T) calculation together with the aug-cc-pVTZ basis set requires large computational effort, the IIEs were calculated only for five representative C⋯C intermolecular bond distances (3.7, 3.8, 3.9, 4.0, and 4.1 Å) around the minimum. In the right graph of Figure 3, one can observe that the LCCSD(T) energies showed an excellent agreement with the reference curve of the CCSD(T) near the energy minimum, with an average deviation of 0.057 kcal/mol. For the CCSD(T)-F12 method, the discrepancies in the energy values were somewhat larger, and we found on average a deviation of 0.258 kcal/mol; the CCSD(T)-F12 method overestimated a bit the conventional CCSD(T) results. This difference between the CCSD(T) and the CCSD(T)-F12 energies may be due to the basis set incompleteness error, which is still present in the energy values obtained with the well-known CCSD(T)/aug-cc-pVTZ level of theory, but counted by the CCSD(T)-F12 through the F12 technique. On the other hand, one should not forget that CCSD(T)-F12 uses the density-fitting and resolution of identity techniques for speeding up the electron integral calculations, the errors of which were also present in the energy difference between the two methods. Accordingly, the real effect of the basis set incompleteness error can be estimated considering the difference between the LCCSD(T) and CCSD(T)-F12 energies (0.2 kcal/mol). If one compares the conventional MP2 results with our reference method (BSSE corrected in both cases), the well-known deficiency of binding energy overestimation for the MP2 method is confirmed again. The mean deviation for MP2 was 0.415 kcal/mol, being the largest among the methods applied for the *B* dimer structure of $CH_{2}Cl_{2}$.

As the next step, we considered theoretical methods at the second order perturbation level including different approximation techniques. The best agreement with the reference method was obtained for SCSN-MP2 with a mean deviation of 0.123 kcal/mol, followed by the LMP2 method with a value of 0.175 kcal/mol. Regarding the DFT methods with different XC functionals, they did not match so well as was found for the perturbation and coupled-cluster methods presented before. Although the discrepancy in the binding energy was not so high, the C⋯C intermolecular bond distance was much shorter than for the earlier investigated cases. At the same time, the long range behavior of the potential curves drawn by the DFT methods was quite different from those obtained with perturbational or coupled-cluster theories.

Summarizing the results presented up to now, one can conclude that the LCCSD(T), SCSN-MP2, and LMP2 results were in a very good agreement with the CCSD(T) reference method. Furthermore, the CCSD(T)-F12 method can also be considered as a solution to reproduce the high level electron correlation effect with an acceptable accuracy. At the same time, the conventional MP2 and the two DFT results were a bit far from the correct description of the full amount of the electron correlation.

As already mentioned, in our previous work [86], we compared the IIEs for the five dimer geometries obtained at different levels of theory. Here, we complete this investigation by introducing the intermolecular energy decomposition scheme defined by the SAPT, as well as by the local electron correlation methods. The complete set of energy values for intermolecular interactions, their dispersion components, and the conformational energy difference of different dimers related to the total energy of structure *A* are presented in Table 1. Schütz et al. [78] pointed out that the dispersion energy derived from the local electron correlation theory was not directly comparable with the SAPT counterpart $E_{disp}^{20}$ (the second-order dispersion energy between two monomers expanded in terms of HF molecular orbitals). They found that the $E_{disp}^{20}$ values were substantially more negative than the dispersion energy defined in the framework of local electron correlation theory. Indeed, comparing the two different dispersion energy results, we can observe on average a shift of 0.6 kcal/mol in favor of the SAPT-type. Therefore, we will not compare these values between them, only show them in the same table. In contrast, the total amounts of the IIEs obtained with the SAPT and local perturbation theories could be compared. Therefore, if one considers the SAPT IIE as the reference energy, the mean absolute deviation (MAD) of the LCCSD(T) energy values was 0.14 kcal/mol, while the similar value for the LMP2 results was 0.16 kcal/mol. Based on this comparison, we can conclude that the intermolecular energy results obtained with different theories were in a good agreement. We performed another comparison for the total dimer energies of the five dimer configurations. In the last row of Table 1, the configuration energy differences are presented. The energy value of the most strongly-bounded dimer structure (Conformation *A*) was chosen as the reference energy. Analyzing the energy differences, one can see that the *A* and *B* structures were very close to each other, with only a 0.08-kcal/mol energy difference. The third structure of *C* was also a strongly-bound dimer; the difference was 0.37 kcal/mol, which is well below the thermal energy (TE) at room temperature (298 K, $E_{k_{B}T}≃0.6$ kcal/mol). The last two structures of *D* and *E* were energetically far from the first two configurations, showing more than a 1-kcal/mol weaker intermolecular bonding.

### 3.2. Methylene Bromide ($CH_{2}Br_{2}$)

The next investigated molecular system was the dibromomethane dimer. Here, we followed the same geometry optimization procedure as in case of the dichloromethane dimer. The equilibrium dimer geometries showed the same spatial symmetries and rank of energies. The IIEs and their dispersion components for the five dimer geometries obtained at different levels of theory, as well as conformational energy differences are presented in Table 2. The HF contribution was either close to zero or repulsive. The energy stability of the dimers was due to the electron correlations. Similar findings were obtained in the case of alkane chains [87], where the HF energy part showed a repulsive behavior, while the electron correlation gave a large attractive contribution. The strongest bounded molecular complexes were the *A* and *B* dimers. Depending on the method used for obtaining the intermolecular energy, the *A* dimer showed a slightly stronger interaction ($\Delta E_{A}^{LMP2}=$ −4.15 kcal/mol and $\Delta E_{B}^{LMP2}=$ −3.98 kcal/mol, as well as $\Delta E_{A}^{LCCSD(T)}=$ −4.00 kcal/mol and $\Delta E_{B}^{LCCSD(T)}=$ −3.86 kcal/mol) in the case of LMP2 or LCCSD(T), while for the SAPT results, the *B* and *A* forms presented almost the same strength ($\Delta E_{B}^{SAPT}=$ −3.85 kcal/mol and $\Delta E_{A}^{SAPT}=$ −4.33 kcal/mol). The nature of interaction is however different: the *A* dimer showed larger repulsion and stronger dispersion attraction than the *B* form. The other three complexes (*C*, *D*, and *E*) showed weaker intermolecular forces, but all their conformational energy differences were close to TE. The *d*(C⋯C) intermolecular bond distance varied between 3.7 and 4.4 Å; the shortest value was taken by the *C* dimer and the largest one by the *D* complex.

**Figure 4:**
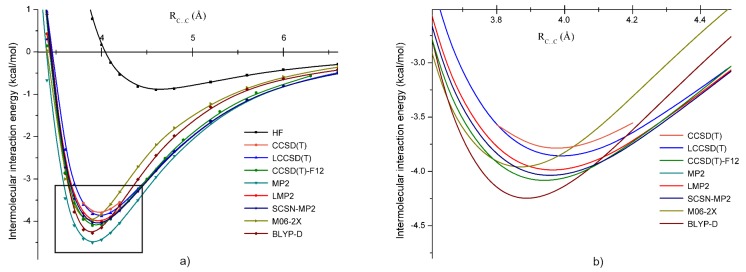
Potential energy curves for the *B* form of the dibromomethane dimer obtained at the HF, CCSD(T), LCCSD(T), CCSD(T)-F12, MP2, LMP2, SCSN-MP2, M06-2X, and BLYP-D levels of theory using the aug-cc-pVTZ basis set; (**a**) for larger intermolcular C⋯C bond separation (2.75–7.25 Å) and (**b**) around the equilibrium C⋯C distance (3.6–4.5 Å).

In order to compare the performance of the applied theoretical methods, we have computed several potential energy curves along the C⋯C line. In Figure 4, we present the potential energy curves obtained at the HF, CCSD(T), LCCSD(T), CCSD(T)-F12, MP2, LMP2, SCSN-MP2, M06-2X, and BLYP-D levels of theory using the aug-cc-pVTZ basis set. In the right side of Figure 4, the curves near the energy minimum are shown in more detail. The CCSD(T) energy was taken again as the reference potential energy curve. First, we compared methods based on the coupled-cluster theory where different theoretical approximations were included. In this case, one can see that energies obtained using localized orbitals (using the LCCSD(T) method) were in a very good agreement with the reference energies. At the energy minimum points, the difference was only 0.07 kcal/mol. The other coupled-cluster method with explicitly-correlated (-F12) approximation overestimated the energy minimum with about 0.27 kcal/mol. Again, this overestimation mostly came from the basis set incompleteness error effects, which was quite similar with the $CH_{2}Cl_{2}$ case (0.2 kcal/mol by taking the energy difference between the CCSD(T)-Fi2 and the LCCSD(T) energies). Almost the same amount of energy deviation was obtained in the previous case of the *B* configuration of the $CH_{2}Cl_{2}$ dimer. The second group of theoretical methods was defined in the framework of MP2 theory. For this case, the canonical MP2 and its local and spin-component scaling approximations were considered. Comparing them with the reference curve, we can see that the canonical MP2 strongly underestimated the reference energy curve, giving energy differences of more than 0.6 kcal/mol. The LMP2 and SCSN-MP2 methods were closer to the reference curve, with a difference of less than 0.2 kcal/mol. Energy curves drawn using the meta-hybrid GGAand the dispersion corrected DFT XC functionals were also in a relatively good agreement with the reference curve. This is especially true for the M06-2X XC functional. However, unlike the previous cases of coupled-cluster and MP2 theories, the DFT minima were shifted by more than 0.1 Å, showing shorter equilibrium bond distances.

### 3.3. Methylene Iodide ($CH_{2}I_{2}$)

For the $CH_{2}I_{2}$ dimer, besides the basis set for the iodine atom, the effective core potential in the case of core electrons was also used [76]. The same geometry optimization procedure was applied (see Section 3.1 and Section 3.2). The optimized dimer geometries showed the same spatial symmetries and rank of energies as for the previous two binary systems. The IIEs, at HF and correlation levels, as well as the C⋯C intermolecular distance and the dispersion part of the IIE for the five dimer geometries of dibromomethane obtained at the DF-LMP2 level of theory are presented in Table 3. The C⋯C distances followed a similar trend as the one found for the $CH_{2}Cl_{2}$ and $CH_{2}Br_{2}$ cases (Table 3). The energy stability of different $CH_{2}I_{2}$ dimer structures was ensured exclusively by the attractive forces at the correlation because HF components for the all five geometries were repulsive. The highest value was obtained for the *C* configuration ($\Delta E_{C}^{HF}=$ 2.86 kcal/mol), where also the *C⋯C* distance was the shortest one, while the *E* dimer geometry showed the “weakest repulsion” ($\Delta E_{C}^{HF}=0.81$ kcal/mol) and the longest *C⋯C* distance. This repulsion was outweighed by the dispersion forces, which showed up at the electron correlation level. The most strongly-bound structure was the *A* geometry ($\Delta E_{A}^{LMP2}=$ −4.82 kcal/mol), followed by the *B* ($\Delta E_{B}^{LMP2}=$ −4.54 kcal/mol), *C* ($\Delta E_{C}^{LMP2}=$ −3.90 kcal/mol), *D* ($\Delta E_{D}^{LMP2}=$ −3.85 kcal/mol), and *E* ($\Delta E_{E}^{LMP2}=$ −3.31 kcal/mol) geometries. The corresponding values obtained with the SAPT theory were: *A* − $\Delta E_{A}^{SAPT}=$ −4.83 kcal/mol, *B* − $\Delta E_{B}^{SAPT}=$ −4.72 kcal/mol, *C* − $\Delta E_{C}^{SAPT}=$ −3.46 kcal/mol, *D* − $\Delta E_{D}^{SAPT}=$ −3.89 kcal/mol, and *E* − $\Delta E_{E}^{SAPT}=$ −3.61 kcal/mol. It can be observed that in the case of *A* and *D* geometries, we had an excellent agreement between the LMP2 and SAPT values, but also for *B* and *E* structures, this difference remained below 0.2 kcal/mol. Only in the *C* case, the difference was larger, 0.44 kcal/mol. The *A* and *B* structures were relatively close to each other ($E_{B-A}^{Conf}$ = 0.25 kcal/mol, while the *C*, *D*, and *E* dimers showed more than 0.9-kcal/mol higher total energy values.

**Figure 5:**
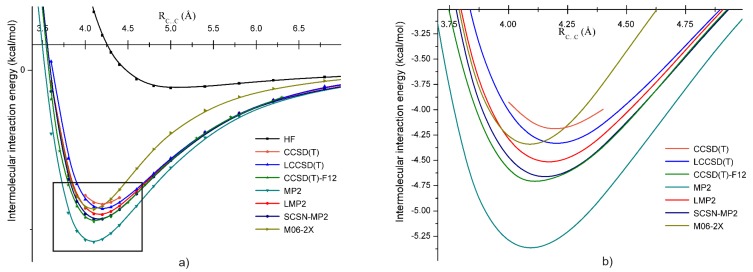
Potential energy curves for the *B* form of the diiodomethane dimer obtained at the HF, CCSD(T), LCCSD(T), CCSD(T)-F12, MP2, LMP2, SCSN-MP2, M06-2X, and BLYP-D levels of theory using the aug-cc-pVTZ basis set. (**a**) for larger intermolcular C···C bond separation (3.25–7 Å) and (**b**) around the equilibrium C···C distance (3.6–5 Å).

Also in this case, we computed potential energy curves along the C⋯C line considering different theoretical models similarly to the previous two cases (see Figure 5). Unfortunately, the BLYP-D calculation could not be performed because of the lack of atomic parameters for iodine atoms in the corresponding quantum chemistry computer code. As seen in Figure 5, left, the HF curve lied very far from the curves obtained with methods where the electron correlation was taken into account, showing that the electron correlation plays an important role in the intermolecular interaction in the $CH_{2}I_{2}$ dimer structure. Considering methods only at the CCSD(T) level of theory, but with different approximation techniques, we can see that the CC method that used localized orbitals (LCCSD(T)) was in a very good agreement with the canonical CCSD(T) results. The difference between the energy minimums was only 0.15 kcal/mol. For the CCSD(T)-F12 method, the deviation was larger, around 0.55 kcal/mol. Accordingly, the basis set incompleteness error can be estimated as the energy difference between the CCSD(T)-F12 and LCCSD(T) energies (∼0.4 kcal/mol), which shows that in the case of the $CH_{2}I_{2}$, the aug-cc-pVTZ basis set was not large enough to cover most of the finite basis set effects. In the case of MP2 quality methods, the best performance was obtained for the LMP2: it gave 0.32 kcal/mol lower energy compared to the reference curve. This was followed by the SCSN-MP2 result, with 0.46 kcal/mol stronger bonding, while the worst approximation was obtained by the canonical MP2 method. It underestimated the reference energy by more than 1.0 kcal/mol. The M06-2X DFT functional gave accurate binding energy, but the equilibrium C⋯C intermolecular distance was shorter by 0.1 Å, and its long-range behavior was different from the others.

### 3.4. Methylene Fluoride ($CH_{2}F_{2}$)

**Figure 6:**
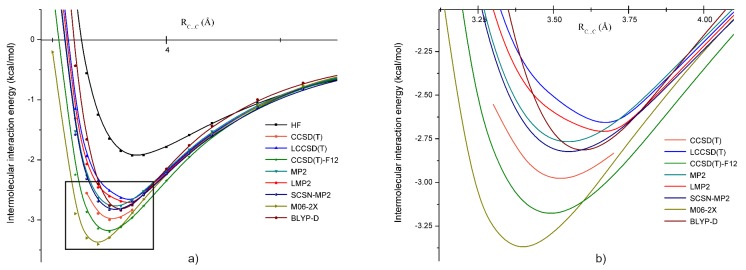
Potential energy curves for the *B* form of the difluoromethane dimer obtained at the HF, CCSD(T), LCCSD(T), CCSD(T)-F12, MP2, LMP2, SCSN-MP2, M06-2X, and BLYP-D levels of theory using the aug-cc-pVTZ basis set; (**a**) for larger intermolcular C⋯C bond separation (3–6 Å) and (**b**) around the equilibrium C⋯C distance (3–4.25 Å).

The intermolecular interaction in the methylene fluoride showed a different picture compared with the other three methylene halides. Only three different dimer structures were obtained, as during the optimization, both the *A* and *C* starting geometries turned over to *B*. The shortest C⋯C intermolecular distance was obtained for the *B* geometry, while the largest one for the *D* structure. The nature of the intermolecular interaction was also different from that in the previous three methylene halide dimers, the *E* structure being energetically more favorable than the *D* one. In this case, we already had a significant binding at the HF level, which was further increased by the electron correlation effects. The HF energies, presented in Table 4, were: $\Delta E_{B}^{HF}=$ −1.75 kcal/mol, $\Delta E_{D}^{HF}=$ −1.02 kcal/mol, and $\Delta E_{E}^{HF}=$ −1.70 kcal/mol, while the total IIE at the correlation level for the LMP2 case were: $\Delta E_{B}^{LMP2}=$ −2.62 kcal/mol, $\Delta E_{D}^{LMP2}=$ −1.67 kcal/mol, and $\Delta E_{E}^{LMP2}=$ −2.08 kcal/mol, and for SAPT, they were: $\Delta E_{B}^{SAPT}=$ −3.47 kcal/mol, $\Delta E_{D}^{SAPT}=$ −1.99 kcal/mol, and $\Delta E_{E}^{SAPT}=$ −2.99 kcal/mol. This binding energy increase was mainly due to the dispersion-type electron correlation effects, both in the LMP2 and SAPT cases. Comparing the LMP2 and SAPT energies, one can observe that the discrepancy between these methods was much larger than for the previous three methylene halides. In order to give an explanation for this discrepancy, one should compare the results with the CCSD(T) potential energy curve around the equilibrium C⋯C intermolecular position (see Figure 6b). The energy minimum obtained with the CCSD(T) method near the equilibrium geometry was 3.0 kcal/mol, which was lower than the LMP2, but higher than the SAPT results (see Figure 6a). Comparison of the potential energy curves with the reference CCSD(T) showed that the MP2, LMP2, SCSN-MP2, and LCCSD(T) methods underestimated, while the CCSD(T)-F12 method overestimated the reference values. It seems that for the case of $CH_{2}F_{2}$, both the basis set incompleteness errors and those induced by the density-fitting and RI approximations were important, but mainly they were canceling each other. Among these methods, the best approximation of the reference energy curves was obtained by the SCSN-MP2 and CCSD(T)-F12 methods, with energy differences near the equilibrium geometry (*d*(C⋯C) = 3.5Å) of +0.17 kcal/mol and −0.20 kcal/mol, respectively. For the DFT methods, the M06-2X overestimated by ∼3.0 kcal/mol, while the BLYP XC functional with empirical dispersion corrections underestimated by ∼0.24 kcal/mol the minimum energy. In both cases, the *d*(C⋯C) equilibrium distance was shifted by ±0.1Å.

### 3.5. Intermolecular Interaction in Methylene Halide Dimers

The range of the intermolecular *d*(C⋯C) distances is given by the relative orientation of the halogen or the hydrogen atoms to each other and thus by the balance of different repulsive and attractive type forces between the halogen and the hydrogen atoms. It was shown by Riley et al. [88] that the electrostatic interaction between the halogen σ-hole and the electronegative halogen bond donor is responsible for the high degree of directionality exhibited by halogen bonds. In our case, we do not have a real halogen σ-hole; there are electrostatic interactions between the electronegative halogen atoms and the electropositive protons. The final electrostatic contribution is the sum of the attractive H⋯X and the repulsive H⋯H and X⋯X pair interactions. On the other hand, these noncovalent interactions have also a strong dispersion component, and therefore, it is important to choose the computational method to treat halogen bonding systems correctly.

**Figure 7:**
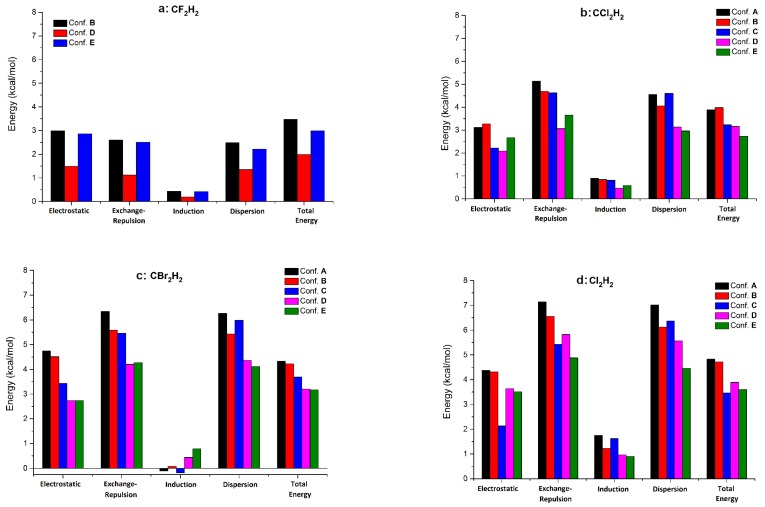
The relative contribution of different energy components (electrostatic, exchange-repulsion, induction, and dispersion) for the four methylene halide dimers (for Conf. A-E see Figure 2).

Each dimer configuration can be coded through the relative positions of the hydrogen and halogen atoms, considering the different numbers of H⋯X pair interactions. In this way, the *A* configuration is given by 2H⋯3X, the *B* by 3H⋯3X, the *C* by 2H⋯4X, the *D* by 2H⋯2X, and the *E* by 4H⋯2X. Of course, as mentioned before, not only the number of H⋯X pair interactions is important, but also the number of X⋯X and H⋯H pair interactions. Comparing the *d*(C⋯C) pair distances in the five dimer structures of dichloro-, dibromo-, and diiodomethane systems, we see that the most compact dimer packing was obtained for the *C* dimer, followed by the *B*, *A*, *D*, and *E* configurations. It is interesting that the conformational and intermolecular energy ranking order did not follow the order of the *d*(*C*⋯*C*) distances. In the case of difluoromethane, the configurations *A* and *C* were missing; therefore, the most compact packing was obtained for the *B* form, followed by the *E* and *D* configurations. Analyzing the different energy contributions to the total intermolecular interaction energy, like electrostatic-, exchange-repulsion-, induction- and dispersion-energy terms, we observed that the total IIEs were obtained as the energy balance of the attractive electrostatic- and dispersion-type and the repulsive exchange-repulsion-type interactions (see Table 5 and Figure 7). The largest contributions were obtained for the exchange-repulsion and dispersion parts, having different signs, followed by the electrostatic contribution and the relatively small induction part. The energy difference between the exchange-repulsion and dispersion parts was in general between −0.5 and +0.6 kcal/mol, mostly canceling each other. There were only two cases—the conformation *E* of dichloromethane and conformation *C* of diiodomethane—where these values were somewhat larger (0.69 kcal/mol and −0.94 kcal/mol, respectively). These results tell us that the strength of the total IIE is determined mainly by the electrostatic contribution, namely the balance of the electrostatic attraction between hetero-atoms (H and halogens) and the electrostatic repulsion between the same type of atoms [89,90]. The relatively small induction contribution added to this electrostatic part can slightly influence the final total interaction energy values. Comparing the total IIEs for all four methylene halides obtained at the local CCSD(T) level of theory, we can conclude that in the gas phase, the most likely conformations for dichloro-, dibromo-, and diiodo-methane dimers are the *A* and *B* forms and for the difluoromethane the *B* form.

## 4. Orientational Correlations in the Liquid Phase

The energy minimum configurations of molecular clusters, obtained by quantum chemical methods, are often useful approximations for the arrangement of the neighboring molecules in the liquid phase. In cases were there are strong specific interactions, such as hydrogen bonding, hydrogen bonded dimers or larger associates are abundant in the liquid phase, as evidenced by diffraction experiments and molecular dynamics simulations. Our molecules lacked such strong characteristic interactions, and the pairwise interaction energies were relatively weak; therefore, the main contribution can be expected to come from vdW forces, and the mutual arrangement and orientation of the close neighbors is likely to be determined by the steric effects. It is interesting to see to what extent the pair interactions determine the mutual arrangement of the closest neighbors in the liquid and if it is possible to learn something about, or predict the mutual orientation, using only ab initio methods for pairs of molecules. Here, we make an attempt to compare the *ab initio* geometries of the dimers, with the most detailed information available to date from diffraction experiments on the corresponding molecular liquids.

Traditionally, the orientational ordering in liquid phase is described by taking into account the angle between two characteristic vectors of the neighboring molecules, and mapping this angle as a function of distance. Recent studies by Pothoczki et al. [91,92] on liquid methylene chloride, bromide, and iodide revealed more detailed features of the mutual orientation of these molecules in the liquid state, not known before. This advance has been achieved by applying a novel geometrical method of analysis of the atomic configurations obtained in force-field computer simulations or neutron and X-ray diffraction experiments combined with reverse Monte Carlo (RMC) simulations.

The most common description of the orientational order for asymmetric molecules, having a unique axis (such as dipole moment), is the variation of the angle between the dipole moments of two molecules with the distance between them. As shown first by Rey [93], substantially more detailed information can be extracted from a three-dimensional atomic model of a liquid, introducing some classification of the mutual orientations of the neighboring molecules. Depending on the shape of the molecule, certain groups can be defined, and the number of molecule pairs belonging to the given group can be calculated. This recipe is not universal, but specific for the geometry of the molecules. For the mutual orientation of tetrahedral molecules, Rey defined six groups, distinguished by the orientation of the closest corner atoms of the pair of molecules, and gave a recipe for calculation using the coordinates of the corner atoms of the tetrahedron [93]. In brief, given a pair of tetrahedral molecules, two parallel planes containing the molecule centers are constructed, and the molecular pairs are classified by the number of the corners belonging to the two molecules, laying between the planes, i.e., looking towards each other. This scheme results in six groups (1:1, corner-to-corner, 1:2, corner-to-edge, and so on.) For molecules with distorted tetrahedral shape, such as methylene halides, or haloforms, the original classification of Rey had been expanded by Pothoczki et al., by introducing several subgroups in which not only the number of atoms between the planes was counted, but also their type was taken into account. For methylene halides, this classification gave 28 subgroups within the six Rey groups, according to the closeness of the given type of atom on one molecule to a distinct atom, side, or edge of the other one [92].

Calculation of the number of pairs of neighbors belonging to a certain group in the simulation boxes can show the most probable mutual orientations in the simulated liquid model.

## 5. Comparison of Nearest Neighbor Orientations in the Liquid and in the Gas Phase

The structure of the difluoromethane dimer in the gas phase has been probed by molecular beam microwave adsorption spectroscopy [20,94]. In both studies, the dimer rotational spectra were found to correspond to the strongest, *B* (face-to-face) dimer configuration, having triple hydrogen bonding structure, with short, improper, or anti-hydrogen bonding. Studies of the heavier methylene halides have not been reported to date. In a recent work, difluoromethane-difluoroethane heterodimers have been studied by pulsed-jet Fourier transform rotational spectroscopy [95]. Interestingly, three dimer configurations could be identified in the spectra, with relative populations following the order of interaction energies calculated at the MP2/6-311++G(3df,3pd) level of theory. The most abundant dimer is a linear one, the $–CHF_{2}$ group of difluoroethane oriented to difluoromethane in the same way as the *B* configuration of the difluoromethane dimer. Larger clusters of three and four $CH_{2}F_{2}$ molecules have also been measured [96,97], and for each case, one computed configuration could be identified in the corresponding rotational spectra [98]. In the trimer, two molecules are oriented to each other like a distorted *D* configuration of the dimers.

As already mentioned, the computed dimers of the three methylene halides look rather similar, and they can be grouped according to the classification of Pothoczki et al. [92], into the following subgroups:

*A*: Y,H - Y,Y,H (edge-to-face)

*B*: H,H,Y - Y,Y,H (face-to-face)

*C*: Y,Y,H - Y,Y,H face-to-face

*D*: Y,Y - H,H edge-to-edge

*E*: H,H,Y - H,H,Y face-to-face

At first glance, it is seen that there are no corner-to-edge or corner-to-face configurations, and that three out of five are face-to-face configurations. It is interesting to look for whether such orientations can be found in the liquid state as well. According to the diffraction data, the first neighbor mutual orientations in the three liquids are dominantly edge-to-face and edge-to-edge, and they account for about 80% of the contact pairs [92]. The lack of corner orientations amongst the dimers is due to the highly unfavorable energy of such configurations, as they would counteract the vdW attraction, as well as to the optimal dipole-dipole orientation. Furthermore, no corner-to-corner neighbors are present in the liquid. The edge-to-edge orientation can be regarded as the intermediate level of “compactness”, situated between the closest face-to-edge/face and the loosest corner-to-corner/edge orientations. Such an arrangement of the molecules in the liquid, in which most of the contacts are of the intermediate compactness, is therefore highly plausible, since the closest orientations for the majority of molecules cannot be simultaneously achieved.

From the analysis of X-ray and neutron scattering measurements of methylene halide molecular liquids [91], Pothoczki et al. summarized the results in a few statements [92]. We cite here (with minor text editing) those that concern the closest neighbor molecular pairs and relate them to the ab initio results for methylene halide dimers.

- *“Dipolar effects, although they are visible, do not have a decisive role in forming pairwise molecular arrangements in $CH_{2}X_{2}$ liquids. Steric effects, on the other hand, are more important.”* Comparing the five calculated configurations, we see that fully-antiparallel dipole orientations appear in Structures *C* and *E*, while a parallel dipole orientation is only seen for Structure *D*. In the remaining two structures with strongest binding, the dipole vectors are nearly perpendicular. Thus, it can be seen that also for the dimers in a vacuum, the dipolar forces are weak, and the attraction of the molecules is rather due to vdW interactions (which are often referred to as steric forces when discussing RMC computer modeling). The weak contribution of the electrostatic forces in molecular liquids $CBrCl_{3}$ and $CHCl_{3}$ was also confirmed in molecular dynamics simulations with Coulomb interactions turned off [99,100].
- *“The most frequent orientation of molecules is of the 2:2 (edge-to-edge) type over the entire distance range in each liquid. Within the 2:2 original group, the H,X-H,X subgroup is the most prominent, apart from the short range orientations in $CH_{2}Cl_{2}$ where the H,H-H,Cl arrangement is the most frequent.”* As mentioned above, we can attribute the dominance of the edge-to-edge orientations to the optimal intermediately-compact arrangement of the molecules. The only 2:2-type mutual orientation occurs in the weakly-bound *D*-type dimer, in which the molecules are aligned with parallel dipole moments, the corresponding subgroup being H,H-X,X. This orientation however does not appear in the liquid, indicating that the dipole interaction is too weak to orient the neighboring molecules.
- *“The structure of liquid methylene chloride appears to be different from the structure of the other two materials. The origin of structural differences is the significant size difference between $CH_{2}Cl_{2}$ and $CH_{2}Br_{2}$ / $CH_{2}I_{2}$ molecules”*.

From the comparisons described above, one can conclude that the mutual orientations of the closest molecules in the liquid rarely adopt one of the five calculated energy minimum configurations. For the studied methylene halide liquids, this is the consequence of the roughly spherical shape of the molecules, for which the interaction energy in the preferred orientations is not much lower than the energies of other mutual orientation. The orientation analysis of the diffraction data also revealed that only a small difference is seen between the actual liquids, as compared to a reference model liquid having tetrahedral molecules with steric repulsion, but no atomic charges.

The situation can by quite different for other molecular liquids, in which the dipole interaction combined with the highly asymmetrical shape of the molecules appreciably determines the mutual orientation of the closest neighbors, such as nitromethane [101] or 1,3,5-trifluorobenzene [102]; in these studies, the diffraction data revealed the existence of the preferred arrangement of the molecular pairs similar to those predicted by ab initio calculations. In hydrogen bonded liquids also, such as water and aqueous solutions, the structure seen by diffraction and simulations usually corresponds rather well to the force-field model predictions.

At low temperature and/or high pressures, methylene halides form crystalline phases [103,104]. Analyzing the crystal packing and cell configurations only, the *D* dimer configuration (head-to-tail) occurs in solid methylene bromide and iodide, while for $CH_{2}Cl_{2}$, none of the *A*-*E* dimer configurations can be recognized in the crystal.

## 6. Conclusions

In this paper, the series of methylene halide ($CH_{2}X_{2}$, X = F, Cl, Br, or I) dimers was studied using various theoretical methods. The conformational energy analysis has shown that in the case of the dichloro-, dibromo-, and diiodo-methane systems, there are two, energetically almost identical conformations (*A* and *B*) with the lowest total energy value. Several dimer conformations, e.g., Conformations *D* and *E* of dichloro- and dibromo-methane or *C* and *D* of the diiodomethane system have quite similar total energy, close to the thermal energy ($k_{B}T$ at room temperature ≈ 0.59 kcal/mol). The largest conformational energy difference of 1.41 kcal/mol indicates that, in the liquid phase at room temperature, all dimer configurations *A*, *B*, *C*, *D*, and *E* can exist with lower or higher probabilities, and none of them can be excluded based on energy considerations. The intermolecular interaction energies of the most stable dimer conformations of different methylene halide dimers are between −3.6 and −4.6 kcal/mol (obtained with the LCCSD(T) method) and are strongly based on electron correlation effects. On the other hand, these electron correlation effects can be surprisingly well covered by the pair-correlation contributions, because the higher order electron correlations give a relatively small contribution to the intermolecular interaction energy. Similar to the saturated hydrocarbons, this intermolecular interaction energy is dominated mainly by the competition of the repulsive exchange and the attractive dispersion energy components, with the difference that in the methylene halide dimers, the electrostatic component has a larger contribution than that found for the saturated hydrocarbons.

The dimer orientations were compared to the closest neighbor orientations observed in the liquid phase in diffraction experiments. Both in liquid, and in vacuo, the dipole-dipole forces were not dominating the mutual interactions. The most frequent orientation observed in the liquid was not very probable in the gas phase, and the strongest dimer was relatively rare in the liquid phase, indicating that for these systems, the molecule pair interactions cannot be simply used to predict the structures expected in the liquid phase. These examples show that for molecular liquids consisting of quasi spherical molecules, more effective theoretical methods such ab initio molecular dynamics simulations or improved ab initio force-fields [105,106] are necessary.

## Figures and Tables

**Table 1:** The intermolecular interaction energies and their dispersion components for the five dimer geometries of dichloromethane obtained at the HF, LMP2, LCCSD(T), and SAPT levels of theory, as well as the conformational energy differences calculated by the LMP2 method (i = B, C, D, E).

Dimer	Unit	A	B	C	D	E
d(C⋯C)	Å	3.91	3.85	3.65	4.09	4.28
$\Delta E^{HF}$	kcal/mol	0.57	−0.04	1.27	−0.70	−1.02
$\Delta E^{LMP2}$	kcal/mol	−3.72	−3.64	−3.34	−2.81	−2.55
$E_{LMP2}^{Disp}$	kcal/mol	−3.86	−3.35	−4.00	−2.53	−2.56
$\Delta E^{LCCSD(T)}$	kcal/mol	−3.58	−3.51	−3.18	−2.72	−2.47
$\Delta E^{SAPT}$	kcal/mol	−3.89	−3.98	−3.23	−3.16	−2.73
$E_{SAPT}^{Disp}$	kcal/mol	−4.56	−4.05	−4.60	−3.13	−2.96
$E_{i-A}^{Conf}$	kcal/mol	-	0.08	0.37	1.15	1.39

**Table 2:** The intermolecular interaction energies and their dispersion components for the five dimer geometries of dibromomethane obtained at the HF, LMP2, LCCSD(T), and SAPT levels of theory, as well as the conformational energy differences calculated by the LMP2 method (i = B, C, D, E).

Dimer	Unit	A	B	C	D	E
d(C⋯C)	Å	4.04	3.96	3.71	4.39	4.25
$\Delta E^{HF}$	kcal/mol	1.17	0.40	1.96	0.62	0.17
$\Delta E^{LMP2}$	kcal/mol	−4.15	−3.98	−3.57	−3.06	−3.01
$E_{LMP2}^{Disp}$	kcal/mol	−4.60	−3.90	−4.63	−3.27	−2.92
$\Delta E^{LCCSD(T)}$	kcal/mol	−4.00	−3.86	−3.40	−2.97	−2.93
$\Delta E^{SAPT}$	kcal/mol	−4.33	−3.85	−3.69	−3.20	−3.17
$E_{SAPT}^{Disp}$	kcal/mol	−6.26	−4.22	−5.98	−4.36	−4.12
$E_{i-A}^{Conf}$	kcal/mol	-	0.14	0.59	1.01	1.07

**Table 3:** The intermolecular interaction energies and their dispersion components for the five dimer geometries of diiodomethane obtained at the HF, LMP2, LCCSD(T), and SAPT levels of theory, as well as the conformational energy differences calculated by the LMP2 method (i = B, C, D, E).

Dimer	Unit	A	B	C	D	E
d(C⋯C)	Å	4.25	4.16	3.80	4.53	4.56
$\Delta E^{HF}$	kcal/mol	2.14	1.36	2.86	1.64	0.81
$\Delta E^{LMP2}$	kcal/mol	−4.82	−4.54	−3.90	−3.85	−3.31
$E_{LMP2}^{Disp}$	kcal/mol	−5.82	−4.97	−5.54	−4.59	−3.59
$\Delta E^{LCCSD(T)}$	kcal/mol	−4.61	−4.34	−3.68	−3.67	−3.17
$\Delta E^{SAPT}$	kcal/mol	−4.83	−4.72	−3.46	−3.89	−3.61
$E_{SAPT}^{Disp}$	kcal/mol	−7.01	−6.12	−6.37	−5.56	−4.46
$E_{i-A}^{Conf}$	kcal/mol	-	0.25	0.92	0.90	1.41

**Table 4:** The intermolecular interaction energies and their dispersion components for the five dimer geometries of difluoromethane obtained at the HF, LMP2, LCCSD(T), and SAPT levels of theory, as well as the conformational energy differences using the aug-cc-pVTZ basis set (i = B, C, D, E).

Dimer	Unit	B	D	E
d(C⋯C)	Å	−3.54	−3.98	−3.61
$\Delta E^{HF}$	kcal/mol	−1.75	−1.02	−1.50
$\Delta E^{LMP2}$	kcal/mol	−2.62	−1.67	−2.08
$E_{LMP2}^{Disp}$	kcal/mol	−1.58	−1.37	−0.90
$\Delta E^{LCCSD(T)}$	kcal/mol	−2.53	−1.63	−1.99
$\Delta E^{SAPT}$	kcal/mol	−3.47	−1.99	−2.99
$E_{SAPT}^{Disp}$	kcal/mol	−2.48	−1.35	−2.21
$E_{i-A}^{Conf}$	kcal/mol	-	0.55	0.87

**Table 5:** SAPT/aug-cc-pVTZ electrostatic (E${}^{ES}$), exchange-repulsion (E${}^{ER}$), induction (E${}^{I}$), and dispersion (E${}^{Disp}$) energy terms for methylene halide ($CH_{2}F_{2}$, $CH_{2}Cl_{2}$, $CH_{2}Br_{2}$, and $CH_{2}I_{2}$) dimers. ΔE${}^{Tot}$ is the total SAPT intermolecular interaction energy and ΔE${}^{HF}$ the total intermolecular interaction energy at the Hartree–Fock level. All energy values are in kcal/mol.

Configuration	Complex	E${}^{\mathrm{ES}}$	E${}^{\mathrm{ER}}$	E${}^{I}$	E${}^{\mathrm{Disp}}$	ΔE${}^{\mathrm{HF}}$	ΔE${}^{\mathrm{Tot}}$
A	CCl${}_{2}$H${}_{2}$	−3.12	5.13	−0.90	−4.56	0.57	−3.89
	CBr${}_{2}$H${}_{2}$	−4.74	6.34	0.11	−6.26	1.17	−4.33
	CI${}_{2}$H${}_{2}$	−4.38	7.14	−1.75	−7.01	2.79	−4.83
B	CF${}_{2}$H${}_{2}$	−2.99	2.60	−0.44	−2.48	−1.75	−3.47
	CCl${}_{2}$H${}_{2}$	−3.27	4.69	−0.84	−4.05	−0.04	−3.98
	CBr${}_{2}$H${}_{2}$	−4.51	5.58	−0.08	−5.43	0.40	−4.22
	CI${}_{2}$H${}_{2}$	−4.31	6.55	−1.23	−6.12	2.27	−4.72
C	CCl${}_{2}$H${}_{2}$	−2.22	4.63	−0.81	−4.60	1.27	−3.23
	CBr${}_{2}$H${}_{2}$	−3.44	5.46	0.18	−5.98	1.96	−3.69
	CI${}_{2}$H${}_{2}$	−2.13	5.43	−1.62	−6.37	3.32	−3.46
D	CF${}_{2}$H${}_{2}$	−1.49	1.12	−0.19	−1.35	−1.02	−1.99
	CCl${}_{2}$H${}_{2}$	−2.08	3.06	−0.46	−3.13	0.23	−3.16
	CBr${}_{2}$H${}_{2}$	−2.74	4.20	−0.44	−4.36	0.62	−3.20
	CI${}_{2}$H${}_{2}$	−3.64	5.82	−0.96	−5.56	2.22	−3.89
E	CF${}_{2}$H${}_{2}$	−2.86	2.50	−0.42	−2.21	−1.50	−2.99
	CCl${}_{2}$H${}_{2}$	−2.67	3.65	−0.58	−2.96	−0.07	−2.73
	CBr${}_{2}$H${}_{2}$	−2.74	4.27	−0.79	−4.12	0.17	−3.17
	CI${}_{2}$H${}_{2}$	−3.51	4.88	−0.90	−4.46	1.39	−3.61
